# Combination of *Helicobacter pylori* infection and the interleukin 8 –251 T > A polymorphism, but not the mannose-binding lectin 2 codon 54 G > A polymorphism, might be a risk factor of gastric cancer

**DOI:** 10.1186/s12885-017-3378-2

**Published:** 2017-05-30

**Authors:** Young Woon Chang, Chi Hyuk Oh, Jung-Wook Kim, Jae Won Lee, Mi Ju Park, Jae-Jun Shim, Chang Kyun Lee, Jae-Young Jang, Seok Ho Dong, Hyo Jong Kim, Sung Soo Kim, Byung-Ho Kim

**Affiliations:** 1 Department of Internal Medicine, Division of Gastroenterology and Hepatology, Kyung Hee University School of Medicine, 23, Kyung Hee Dae-ro, Dongdaemun-gu, Seoul, Seoul 02447 South Korea; 2 Department of Statistics, Korea University, Seoul, South Korea; 3 Department of Biochemistry and Molecular Biology, Kyung Hee University School of Medicine, Seoul, South Korea

**Keywords:** Mannose-binding lectin 2, Interleukin 8, *Helicobacter pylori*, Gastric cancer

## Abstract

**Background:**

Mannose-binding lectin (MBL) acts in the innate immune response to *Helicobacter pylori*. Interleukin 8 (IL-8) is a potent cytokine produced by gastric epithelial cells in response to *H. pylori*. We aimed to investigate whether polymorphisms in *MBL2* and *IL-8* influence susceptibility to *H. pylori* infection, and the associations of these polymorphisms with the risk of gastroduodenal diseases in a Korean population.

**Methods:**

We consecutively enrolled 176 *H. pylori*-negative control subjects, 221 subjects with *H. pylori*-positive non-atrophic gastritis, 52 mild atrophic gastritis (AG), 61 severe AG, 175 duodenal ulcer, and 283 gastric cancer (GC). Allele-specific PCR-RFLP was conducted for polymorphisms in *MBL2* exon 1 (codon 52, 54, and 57) and *IL-8* -251 T > A. IL-8 levels in gastric mucosal tissues and serum MBL levels were measured by enzyme-linked immunosorbent assay.

**Results:**

*MBL2* exon 1 polymorphic variants were found only in codon 54, and the allele frequencies did not differ significantly between the control and disease groups. Although serum MBL levels in codon 54 *A/A* mutants were markedly low, it did not influence susceptibility to *H. pylori* infection or the risk of gastroduodenal diseases. IL-8 levels were significantly different between *T/T* wild type, *T/A* heterozygote, and *A/A* mutant genotypes. *IL-8* -251 *A* allele carriers (*A/A* + *T/A*) showed increased IL-8 levels, and were significantly associated with the risk of severe AG and GC.

**Conclusions:**

We suggest that a combination of *H. pylori* infection and the *IL-8* -251 T > A polymorphism might increase the risk of severe AG and GC in a Korean population.

## Background

The *Helicobacter pylori* infection rate is about 50% among the worldwide adult population [1]. In Korea, the adult *H. pylori* infection rate was 66.9% in 1998, 59.6% in 2005, and dropped to 54.4% in 2011 [2]. The main cause of this decrease in infection rate is the improvement in unsanitary environmental conditions. Besides environmental factors, bacterial and host factors are involved in the pathogenesis of *H. pylori* infection. With regard to bacterial factors, *H. pylori* strains possessing the virulence factors cagA, vacA s1a/m1, and iceA1 are known to be particularly virulent, and are frequently associated with severe gastric epithelial damage [3]. In contrast to Western populations, the cagA protein is commonly found in Korean patients with gastric cancer (GC) and duodenal ulcer (DU) [4]. However, there have been no associations reported between different *H. pylori* genotypes and clinical outcome in Korean patients [5, 6].

With regard to host factors, host genetic variants may influence susceptibility to *H. pylori* and the pathogenesis of gastroduodenal diseases. Host factors are mainly related to two processes: recognition of *H. pylori* by the innate immune system, and the level of the cytokine response [7, 8]. Polymorphisms in pro- and anti-inflammatory cytokines are associated with the risk of atrophic gastritis (AG) and GC. Interleukin 1 beta (IL-1β), tumor necrosis factor-alpha, IL-6, and IL-8 are up-regulated in response to *H. pylori* infection [9]. Several anti-inflammatory cytokines such as IL-Rα and IL-10 are also related to *H. pylori* infection [10, 11].

IL-8 is a major neutrophil-activating cytokine and plays a central role in the immuno-pathogenesis of *H. pylori*-induced gastric mucosal injury. IL-8 levels are 10-fold higher in GC specimens than in normal gastric tissues [12]. The *IL-8* -251 T > A polymorphism has been reported to be associated with increased production of IL-8 protein, and higher risks of AG, gastric ulcer, and GC [13–17]. However, many other reports are inconsistent with these findings [18–23], and a meta-analysis of epidemiological studies revealed no overall association [24].

The innate immune response to *H. pylori* infection is a further candidate host factor. Toll-like receptors (TLRs) recognize conserved pathogen-associated molecular patterns expressed by many pathogens, including *H. pylori* [25]. Mannose-binding lectin (MBL), a pattern recognition receptor encoded by the *MBL2* gene, recognizes lipopolysaccharide in the cell wall of gram-negative bacteria such as *H. pylori* [26, 27]. *H. pylori* activates MBL in vitro, resulting in complement deposition [28, 29]. Some studies have found a possible association of *MBL2* haplotype with susceptibility to *H. pylori* infection, as well as with risk of GC [30, 31]. However, other studies did not find any significant association between *MBL* genotype and *H. pylori* infection prevalence or GC risk [32, 33].

Serum MBL levels vary widely between healthy individuals, mainly due to genetic variation [34–36]. The variation in serum MBL levels is correlated with point mutations in the coding and promoter regions of *MBL2*. Three mutations within exon 1 (in codons 52, 54, and 57) interfere with MBL function and are associated with low serum levels of MBL. In African populations, point mutations at codons 52 and 57 occur frequently [36, 37]. In Caucasians, mutations at codons 52 and 54 are common [38]. In Chinese, Japanese, and Korean populations, mutations are predominantly common in codon 54, but not in codons 52 or 57 [39–41]. Polymorphisms within the promoter and 5′-untranslated regions of *MBL2* also affect serum levels of MBL, but the effects were found to be lower than those of the exon 1 polymorphisms [41].

The aims of this study were: 1) to examine the influence of the polymorphisms in codons 52, 54, and 57 of *MBL2* (related to innate immunity) on susceptibility to *H. pylori* infection; 2) to evaluate the association of the *IL-8* -251 T > A polymorphism with the risk of gastroduodenal diseases in a Korean population; and 3) to analyze our and other investigators’ large-scale data regarding the *IL-8* -251 T > A polymorphism and GC risk in Korean, Japanese, Chinese, and Caucasian populations.

## Methods

From January 2012 to May 2015, *H. pylori*-negative healthy control subjects (control, *n* = 176), *H. pylori*-positive non-atrophic gastritis patients (NAG, *n* = 108), *H. pylori*-positive mild AG patients (*n* = 52), *H. pylori*-positive severe AG patients (*n* = 61), DU patients (*n* = 175), and GC patients (*n* = 283) were consecutively enrolled.

All participants (*n* = 855) underwent upper gastrointestinal endoscopy and routine laboratory tests. The controls were asymptomatic subjects who visited the Health Screening Center for a health status check-up, and their endoscopic findings were normal. Exclusion criteria were *H. pylori* eradication history; use of antibiotics, proton pump inhibitors, nonsteroidal anti-inflammatory drugs, or anticoagulant drugs; and severe systemic illnesses. Age, sex, alcohol consumption (current or never), smoking habits (current or never), salt intake (high, low-moderate), and family history of GC (first-degree relatives) were recorded. Informed consent was obtained from all included subjects. The Institutional Review Board of the Kyung Hee University Hospital approved the study protocol (KMC IRB 1523–04).

### Diagnosis of *H. pylori* infection

The rapid urease test (or urea breath test) and serum anti-*H. pylori* immunoglobulin G antibody test were performed. A subject was defined as *H. pylori* infection-positive if both tests were positive. A subject was defined as *H. pylori* infection-negative if both tests were negative. Subjects with only one positive test were excluded from this study.

### Histologic examination of chronic gastritis

One pathologist histologically evaluated chronic gastritis status in biopsy specimens. AG was graded based on the presence and proportion of glandular loss (mild, moderate, and severe) according to the updated Sydney system [42].

### Genotyping of *MBL2* exon 1 codons 52, 54 and 57, and of *IL-8* -251

Genomic DNA was extracted from peripheral venous blood using a genomic DNA purification method. Polymerase chain reaction (PCR) amplification, restriction fragment length polymorphism (RFLP) analysis, and gel electrophoresis were performed for *MBL2* (codons 52, 54, and 57 in exon 1) and *IL-8* (−251 promoter region) as described previously [7, 34]. The PCR product involving codon 52 was digested by incubation with *Mlu*I at 37 °C for 3 h, resulting in two bands of 204 and 94 bp for the T/T genotype (mutant), three bands of 298, 204, and 94 bp for the A/T genotype (heterozygote), and one band of 298 bp for the A/A genotype (wild type). The PCR product involving codon 54 was digested by *Ban*I at 50 °C for 3 h, resulting in two bands of 195 and 103 bp for the G/G genotype (wild type), three bands of 298, 195, and 103 bp for the G/A genotype (heterozygote), and one band of 298 bp for the A/A genotype (mutant). The PCR product involving codon 57 was digested with *Mbo*I at 37 °C for 3 h, resulting in two bands of 190 and 108 bp for the A/A genotype (mutant), three bands of 298, 190, and 108 bp for the G/A genotype (heterozygote), and one band of 298 bp for the G/G genotype (wild type). For genotyping of the *IL-8* -251 T > A polymorphism, PCR products were digested with *MfeI* at 37 °C for 3 h, resulting in two bands of 449 and 92 bp for the A/A genotype (mutant), three bands of 541, 449, and 92 bp for the T/A genotype (heterozygote), and one band of 541 bp for the T/T genotype (wild type).

### Measurement of serum MBL levels

MBL is a serum protein produced mainly by hepatocytes, and expressed in immune cells, but not in epithelial cells [43]. Circulatory MBL levels were taken as an indicator of the functional activity of MBL protein. Serum MBL levels were measured after overnight fasting by enzyme-linked immunosorbent assay (ELISA; MBL Oligomer ELISA kit; BioProto Diagnostics, Denmark).

### Measurement of IL-8 levels in gastric mucosal tissues

Although measurement of serum IL-8 levels is straightforward, serum IL-8 levels do not reflect the severity of *H. pylori*-associated gastritis [44]. Therefore, we measured IL-8 levels in gastric mucosal tissues rather than serum IL-8 levels.

Three biopsy specimens were taken from the greater curvature side of the proximal antrum during endoscopic procedures. The specimens were put into a tube with 2.0 mL phosphate-buffered saline (pH 7.4), frozen on dry ice, and stored at −70 °C. Samples were homogenized and centrifuged, and the supernatants were aliquoted. Total protein was measured using the bicinchoninic acid assay (Thermo Scientific, Rockford, IL, USA). Gastric mucosal IL-8 levels were measured by ELISA (R&D Systems Inc., Minneapolis, MN, USA). The mucosal level of IL-8 was expressed as picograms per milligram of gastric biopsy protein.

### Analysis of global raw data regarding IL-8 -251 T > A polymorphism and GC risk

The results obtained regarding the association of GC risk with *IL-8* -251 T > A genotype was not consistent with previous epidemiological results [18–24]. Therefore, we collected large-scale raw data of GC patients (*n* = 3217) and controls (*n* = 3810) from Asian (Korea, Japan, and China), and Caucasian (Poland, Finland, and Portugal) populations [13–23], and analyzed GC risk according to *IL-8* -251 T > A genotype.

### Statistical analysis

Data are expressed as mean values ± standard deviations or as frequencies and percentages. Chi-squared and Kruskal–Wallis tests were performed to compare clinical parameters between the control and disease groups. Hardy–Weinberg equilibrium for polymorphisms in *MBL2* and *IL-8* was tested using R version 3.1.0 (R Development Core Team). Biases caused by differences in clinical parameters were adjusted using the chi-squared and Kruskal–Wallis tests. Multiple logistic regression analysis was performed to evaluate the associations of the genetic polymorphisms with susceptibility to *H. pylori* infection and the risk of gastroduodenal diseases using the SAS statistical software package version 9.4 (SAS Institute Inc.). All clinical parameters with a *p* value <0.20 in the univariate analysis were included in the full logistic regression model. The odds ratios (ORs) and their 95% confidence intervals (CIs) were used to compare the risks between the control and disease groups. *P* values <0.05 were considered statistically significant.

## Results

Table 1 shows clinical features of the control and disease groups. Age, sex ratio, and alcohol consumption were similar among all groups. Risk factors for GC, such as smoking, high salt intake, and family history of GC, were more frequently observed in the *H. pylori*-positive GC group than in the control group. The differences were statistically significant.

**Table 1:** Basic clinical features of the control and disease groups

	Hp (−) Control (*n* = 176)	Hp (+) NAG (*n* = 108)	Hp (+) Mild AG (*n* = 52)	Hp (+) Severe AG (*n* = 61)	DU (*n* = 175)	GC (*n* = 283)	*p*
Sex (M:F)	87:89	41:67	23:29	37:24	90:85	151:132	0.1832^a^
Mean age (years)	52.5 ± 6.7	51.58 ± 6.65	59.52 ± 8.30	60.89 ± 8.55	53.2 ± 12.6	55.5 ± 8.8	0.1431^b^
Alcohol consumption (%)	52.27%	51.85%	48.46%	47.54%	57.71%	55.48%	0.1751^a^
Smoking habits (%)	19.89%	18.52%	30.77%	26.23%	58.86%	40.99%	<0.0001^a^
High salt intake (%)	25.00%	26.85%	16.92%	49.18	39.43%	48.41%	<0.0001^a^
FHx of GC (%)	10.23%	15.52%	13.79%	16.39%	10.86%	20.85%	0.0043^a^
Hp positivity (%)	0%	100%	100%	100%	96.00%	73.14%	<0.0001^a^

a: Chi square test for comparison of percentage (GC vs control)

b: Kruskal-Wallis test for comparison of mean ± SD (GC vs control)

The frequencies of the *MBL2* codon 54 and *IL-8* -251 polymorphisms in the control group did not deviate significantly from those expected under Hardy–Weinberg equilibrium (*p* = 1.000 and *p* = 0.184, respectively). In this study population, *MBL* exon 1 polymorphic variants were found only in codon 54. There were no variants at codons 52 or 57; only the wild type was observed.

### The frequencies of *MBL2* codon 54 and *IL-8 -*251 genotypes in the control and disease groups

The frequencies of *MBL2* codon 54 genotypes were similar among the control and disease groups (Table 2). The frequency of *IL-8* -251 A allele carriers was higher in the *H. pylori*-positive severe AG and *H. pylori*-positive GC groups than in the control group, but the differences did not reach statistical significance (Table 2).

**Table 2:** The frequencies of *MBL2* codon 54 and *IL-8 -*251 genotypes in the control and disease groups

Genotype		Control *n* = 176 (%)	Hp (+) NAG *n* = 108 (%)	Hp (+) Mild AG *n* = 52 (%)	Hp (+) Severe AG *n* = 61 (%)	Hp (−) DU *n* = 7 (%)	Hp (+) DU *n* = 168 (%)	Hp (−) GC *n* = 76 (%)	Hp (+) GC *n* = 207 (%)
*MBL2* codon 54	G/G (wild type)	103 (58.52)	65 (60.19)	29 (55.77)	32 (52.46)	4 (57.14)	95 (56.55)	41 (53.95)	129 (62.32)
	G/A(heterozygote)	63 (35.80)	37 (34.26)	22 (43.31)	27 (44.26)	3 (42.86)	69 (41.07)	29 (32.37)	67 (32.37)
	A/A (mutant)	10 (5.68)	6 (5.56)	1 (1.92)	2 (3.28)	0 (0.00)	4 (2.38)	6 (5.31)	11 (5.31)
	G/A + A/A (A carrier)	73 (41.48)	43 (39.81)	23 (44.23)	29 (47.54)	4 (57.14)	73 (43.45)	35 (46.05)	78 (37.68)
*IL-8* -251	T/T (wild type)	70 (39.77)	52 (48.15)	20 (38.46)	15 (24.59)	3 (42.86)	81 (48.21)	23 (30.26)	58 (28.02)
	T/A (heterozygote)	89 (50.57)	49 (45.37)	27 (51.92)	37 (60.66)	3 (42.86)	73 (43.45)	46 (60.53)	122 (58.94)
	A/A (mutant)	17 (9.66)	7 (6,48)	5 (9.62)	9 (16.75)	1 (14.29)	14 (8.33)	7 (9.21)	27 (13.04)
	T/A + A/A (A carrier)	106 (60.23)	56 (51.85)	32 (61.54)	46 (75.41)	4 (57.14)	87 (51.79)	53 (69.74)	149 (71.99)

The frequencies of *MBL2* codon 54 and *IL-8* -251 genotypes were not significantly different between the control and disease groups

### Association between *MBL2* codon 54 G > A polymorphism and the risk of gastroduodenal diseases

We examined the association between the *MBL2* codon 54 G > A polymorphism and the risk of gastroduodenal disease using univariate and multivariate logistic regression analysis. We regarded the control group as the reference subject group, and considered G/G (wild type) as the reference genotype. The *MBL2* codon 54 G > A polymorphism did not increase susceptibility to *H. pylori*-positive NAG, mild AG, or severe AG, and also was not associated with the risk of DU and GC (Table 3).

**Table 3:** MBL codon 54 G > A polymorphism and the risk of gastroduodenal diseases

Genotype	Disease group	Unadjusted OR (95% CI) by univariate analysis	*p*	Adjusted OR (95% CI) by multivariate analysis	*p*
G/A (heterozygote)	Hp(+) NAG (*n* = 49)	0.93 (0.56–1.55)	0.7828	1.02 (0.59–1.75)	0.9466
	Hp(+) mild AG (*n* = 27)	1.24 (0.66–2.34)	0.5074	1.16 (0.60–2.26)	0.6527
	Hp(+) severe AG (*n* = 37)	1.38 (0.76–2.52)	0.2937	1.16 (0.61–2.18)	0.6498
	Hp(−) DU(*n* = 3)	1.23 (0.27–5.66)	0.7938	1.21 (0.26–5.71)	0.8082
	Hp(+) DU (*n* = 73)	1.19 (0.76–1.85)	0.4450	1.24 (0.78–1.99)	0.3605
	Hp(−) GC(*n* = 46)	1.16 (0.65–2.04)	0.6170	1.11 (0.61–2.00)	0.7356
	Hp(+) GC (*n* = 122)	0.85 (0.55–1.31)	0.4566	0.85 (0.54–1.34)	0.7356
A/A (mutant)	Hp(+) NAG (*n* = 7)	0.95 (0.33–2.74)	0.9255	1.19 (0.39–3.61)	0.7561
	Hp(+) mild AG (*n* = 5)	0.36 (0.04–2.89)	0.3332	0.33 (0.04–2.81)	0.3126
	Hp(+) severe AG (*n* = 9)	0.64 (0.13–3.09)	0.5822	0.61 (0.12–3.12)	0.5560
	Hp(−) DU (*n* = 1)	1.25(0.32–5.88)	0.9521	1.24(0.31–5.77)	0.9434
	Hp(+) DU (*n* = 14)	0.43 (0.13–1.43)	0.1697	0.45 (0.13–1.55)	0.2031
	Hp(−) GC (*n* = 7)	1.51 (0.52–4.42)	0.4543	1.93 (0.63–5.89)	0.2484
	Hp(+) GC (*n* = 27)	0.88 (0.36–2.15)	0.7762	1.10 (0.43–2.79)	0.8496
G/A + A/A (A carrier)	*Hp(+) NAG (n = 56)*	0.93 (0.57–1.52)	0.7820	1.04 (0.62–1.75)	0.8774
	Hp(+) mild AG (*n* = 32)	1.12 (0.60–2.09)	0.7239	1.05 (0.55–2.00)	0.8893
	Hp(+) severe AG (*n* = 46)	1.28 (0.71–2.29)	0.4103	1.09 (0.59–2.01)	0.7929
	Hp(−) DU (*n* = 4)	1.06 (0.23–4.87)	0.9421	1.06 (0.23–4.98)	0.9386
	Hp(+) DU (*n* = 87)	1.08 (0.71–1.66)	0.7110	1.13 (0.72–1.78)	0.5868
	Hp(−) GC (*n* = 53)	1.20 (0.70–2.07)	0.5008	1.19 (0.68–2.09)	0.5480
	Hp(+) GC (*n* = 149)	0.85 (0.57–1.29)	0.4488	0.88 (0.57–1.35)	0.5528

The control was regarded as the reference subject group, and the G/G wild type was considered as the reference genotype

### Serum MBL levels

**Fig. 1:**
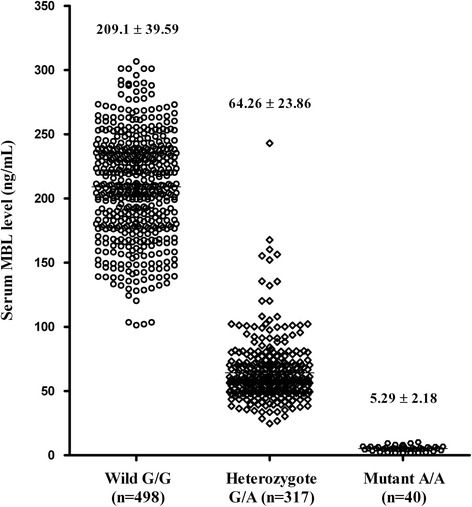
Serum MBL levels in all subjects according to *MBL2* codon 54 genotype. Serum MBL levels differed significantly between the three genotypes, as determined by the Kruskal–Wallis test (*p* < 0.0001)

Serum levels of MBL were high in carriers of the G/G (wild type) genotype, intermediate in those with the G/A heterozygous genotype, and low in those with the A/A (mutant) genotype in all subjects (*n* = 855, Fig. 1). The differences between the three genotypes were highly statistically significant (*p* < 0.0001). However, there were no significant differences in serum MBL levels between the control (139.9 ± 83.2 ng/mL), *H. pylori*-positive NAG (149.3 ± 81.2 ng/mL), mild AG (146.9 ± 81.8 ng/mL), severe AG (140.2 ± 87.3 ng/mL), DU (143.8 ± 82.5 ng/mL), and GC (149.8 ± 82.6 ng/mL) groups.

### Association between IL-8 -251 T > A polymorphism and the risk of disease development

Because the IL-8 cytokine response is mainly dependent on the *H. pylori*-associated inflammatory severity, we sub-classified the *H. pylori*-positive chronic gastritis group (*n* = 221) into *H. pylori*-positive NAG, mild AG, and severe AG. We regarded the control group as the reference subject group, and considered T/T (wild type) as the reference genotype. The *IL-8* -251 A allele significantly increased the risk of severe AG and GC, as determined by both univariate and multivariate logistic regression analysis (Table 4).

**Table 4:** *IL-8* -251 T > A polymorphism and the risk of gastroduodenal diseases

Genotype	Disease group	Unadjusted OR (95% CI) by univariate analysis	*p*	Adjusted OR (95% CI) by multivariate analysis	*p*
T/A (heterozygote)	Hp(+) NAG (*n* = 49)	0.74 (0.45–1.22)	0.2406	0.76 (0.46–1.29)	0.3018
	Hp(+) mild AG (*n* = 27)	1.06 (0.55–2.05)	0.8581	1.09 (0.55–2.16)	0.8052
	Hp(+) severe AG (*n* = 37)	1.94 (0.99–3.82)	0.0549	2.06 (1.01–4.21)	0.0471
	Hp(−) DU(*n* = 3)	0.79 (0.15–4.02)	0.7729	0.84 (0.16–4.36)	0.8342
	Hp(+) DU (*n* = 73)	0.71 (0.45–1.11)	0.1297	0.80 (0.50–1.29)	0.3616
	Hp(−) GC(*n* = 46)	1.57 (0.87–2.84)	0.1326	1.66 (0.89–3.07)	0.1058
	Hp(+) GC (*n* = 122)	1.65 (1.06–2.58)	0.0257	1.71 (1.07–2.72)	0.0252
A/A (mutant)	Hp(+) NAG (*n* = 7)	0.55 (0.21–1.43)	0.2237	0.59 (0.21–1.59)	0.2952
	Hp(+) mild AG (*n* = 5)	1.03 (0.34–3.14)	0.9593	1.00 (0.31–3.17)	0.9941
	Hp(+) severe AG (*n* = 9)	2.47 (0.93–6.59)	0.0710	2.42 (0.85–6.84)	0.0966
	Hp(−) DU (*n* = 1)	1.37 (0.13–14.03)	0.7895	1.66 (0.16–17.71)	0.6743
	Hp(+) DU (*n* = 14)	0.71 (0.33–1.55)	0.3905	0.72 (0.32–1.63)	0.4289
	Hp(−) GC (*n* = 7)	1.25 (0.46–3.40)	0.6577	1.19 (0.43–3.34)	0.7403
	Hp(+) GC (*n* = 27)	1.92 (0.95–3.86)	0.0683	1.94 (0.93–4.06)	0.0744
T/A + A/A (A carrier)	Hp(+) NAG (*n* = 56)	0.71 (0.44–1.15)	0.1669	0.73 (0.44–1.22)	0.2282
	Hp(+) mild AG (*n* = 32)	1.06 (0.56–1.99)	0.8651	1.07 (0.55–2.08)	0.8336
	Hp(+) severe AG (*n* = 46)	2.03 (1.05–3.90)	0.0351	2.12 (1.06–4.25)	0.0335
	Hp(−) DU (*n* = 4)	0.88 (0.19–4.05)	0.8703	0.96 (0.20–4.48)	0.3066
	Hp(+) DU (*n* = 87)	0.71 (0.46–1.09)	0.1152	0.79 (0.50–1.24)	0.3035
	Hp(−) GC (*n* = 53)	1.52 (0.86–2.70)	0.1524	1.58 (0.87–2.87)	0.1346
	Hp(+) GC (*n* = 149)	1.70 (1.11–2.60)	0.0155	1.74 (1.11–2.74)	0.0161

The control was regarded as the reference subject group, and the T/T wild type was considered as the reference genotype

### Comparison of IL-8 levels according to disease phenotypes and IL-8 -251 each genotype

**Fig. 2:**
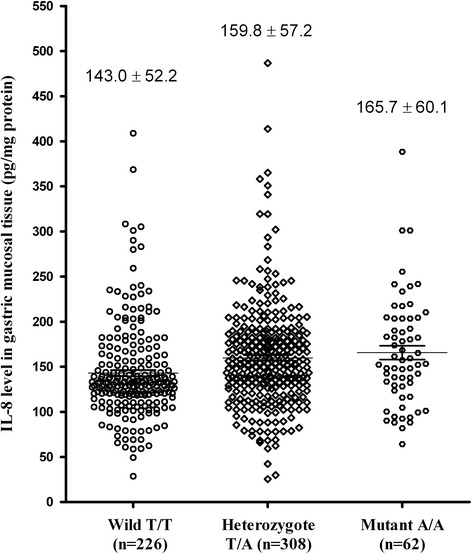
IL-8 levels in all *H. pylori*-positive subjects according to *IL-8* -251 genotype. IL-8 levels differed significantly between the three genotypes, as determined by the Kruskal–Wallis test (*p* = 0.0262)

IL-8 levels were low in subjects with the T/T (wild type) genotype, intermediate in those with the T/A heterozygous genotype, and high in those with the A/A (mutant) genotype. The differences between the three genotypes were statistically significant (*p* = 0.0262, Fig. 2).

**Fig. 3:**
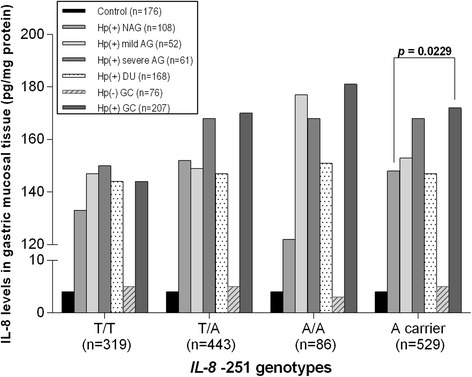
IL-8 levels according to disease phenotypes and *IL-8* -251 genotype. The disease groups did not show any significant differences in IL-8 levels between the T/T, T/A, and A/A genotypes or for A allele carriers, as determined by multiple group comparison test. However, *H. pylori*-positive GC A allele carriers showed significantly higher IL-8 levels than NAG A allele carriers (*p* = 0.0229), as determined by two group comparison test

IL-8 levels were markedly low in *H. pylori*-negative control (*n* = 176, 4.43 ± 3.30 pg/mg protein) and *H. pylori*-negative subjects regardless of any disease phenotypes (*n* = 83, 5.02 ± 3.80 pg/mg protein) compared with *H. pylori*-positive subjects (*n* = 596, 154.05 ± 56.26 pg/mg protein; *p* < 0.0001). IL-8 levels were very low in the *H. pylori*-negative DU and *H. pylori*-negative GC groups as well as in the control group. Therefore, we regarded the *H. pylori*-positive NAG group as the reference subject group instead of the control group. We selected five *H. pylori*-positive disease groups to evaluate the gastric precancerous cascade: NAG, mild AG, severe AG, DU, and GC (Fig. 3). The five disease groups did not show any significant differences in IL-8 levels for the T/T (*p* = 0.7979), T/A (*p* = 0.2200), or A/A (*p* = 0.1000) genotypes, or A allele carriers (*p* = 0.0550), as analyzed by a multiple group comparison test. However, *H. pylori*-positive GC A allele carriers (*n* = 207, 172.3 ± 65.4 pg/mg protein) showed significantly higher IL-8 levels than NAG A allele carriers (*n* = 108, 148.3 ± 42.9 pg/mg protein, *p* = 0.0229), as determined by the two-group comparison test (Fig. 3).

### Analysis of global results of *IL-8* -251 T > A polymorphism and GC risk

The Korean population, including the subjects of this study, showed a significant positive association between the *IL-8* -251 T > A polymorphism and GC risk. On the contrary, the Chinese and Caucasian populations showed a negative association. The Japanese population was similar to the Korean population. The combined Korean and Japanese populations showed significantly increased GC risk for the *IL-8* -251 T/A and A/A genotypes compared to the T/T genotype, and for A allele carriers compared to non-carriers (Table 5).

**Table 5:** The association of *IL-8* -251 T > A polymorphism with the risk of GC in different ethnicities

Ethnicity	TT / total GC (%) TT / total controls (%) OR [95% CI]	TA / total GC (%) TA / total controls (%) OR [95% CI]	AA / total GC (%) AA / total controls (%) OR [95% CI]	A* / total GC (%) A* / total controls (%) OR [95% CI]
This study (Korean)	81/283 (28.62%) 70/176 (39.77%) 0.60 [0.40, 0.90]	168/283(59.36%) 89/176 (50.57%) 1.43 [0.98, 2.09]	34/283(12.01%) 17/176(9.66%) 1.27 [0.70, 2.31]	202/283(71.38%) 106/176 (60.23%) 1.66 [1.11, 2.47]
Korean [16, 17]	180/487(36.96%) 244/528(46.21%) 0.68 [0.53, 0.88]	241/487(49.49%) 234/528(44.32%) 1.23 [0.96, 1.57]	66/487(13.55%) 50/528(9.47%) 1.50 [1.02, 2.20]	307/487(63.04%) 284/528 (53.79%) 1.46 [1.14, 1.88]
Japanese [13–15]	337/789(42.71%) 485/964(50.31%) 0.74 [0.61, 0.89]	375/789(47.53%) 397/964(41.18%) 1.29 [1.07, 1.56]	77/789(9.76%) 82/964(8.51%) 1.16 [0.84, 1.62]	452/789(57.29%) 479/964(49.69%) 1.36 [1.12, 1.64]
Chinese [18–20]	329/926(35.53%) 270/814(33.17%) 1.11 [0.91, 1.35]	425/926(45.90%) 406/814(49.88%) 0.85 [0.71, 1.03]	172/926(18.57%) 138/814(16.95%) 1.12 [0.87, 1.43]	597/926(64.47%) 544/814(66.83%) 0.90 [0.74, 1.10]
Caucasian [21–23]	224/732(30.60%) 381/1328(28.69%) 1.10 [0.90, 1.34]	365/732(49.86%) 669/1328(50.38%) 0.98 [0.82, 1.17]	143/732(19.54%) 278/1328(20.93%) 0.92 [0.73, 1.15]	508/732(69.40%) 947/1328(71.31%) 0.91 [0.75, 1.11]
Combined Korean and Japanese	598/1559(38.36%) 799/1668(47.90%) 0.70 [0.61, 0.81]	784/1559(50.29%) 720/1668(43.17%) 1.29 [1.12, 1.48]	177/1559(11.35%) 149/1668(8.93%) 1.29 [1.02, 1.62]	961/1559(61.64%) 869/1668(52.10%) 1.42 [1.24, 1.64]

A*: A allele carriers (T/A + A/A)

The combined Korean and Japanese populations showed significant GC risk in *IL-8* -251 T/A, A/A, and A allele carriers

## Discussion

The innate immune response is the first line of defense against *H. pylori* infection in the human stomach. TLR and MBL are recognized as important proteins in innate immunity. Several studies have demonstrated that *TLR4* and *TLR2* polymorphisms are associated with the risk of GC [45–47]. However, some of the associations are controversial, and there are discrepancies between the results for Asian and Western populations [48]. A recent study in the Netherlands found that only the *TLR1* polymorphism is associated with the prevalence of *H. pylori* seropositivity [49]. Further studies are needed in other populations worldwide to confirm these associations.

MBL binds to bacteria, yeasts, and viruses via specific repeated oligosaccharide moieties on the cell surface. MBL activates the complement-lectin pathway, facilitates opsonization and phagocytosis, and induces direct cellular lysis. MBL deficiency or a low serum MBL level has been associated with several infectious and autoimmune diseases, including meningococcal meningitis, pneumonia, arterial thrombosis, systemic lupus erythematosus, and celiac disease [50, 51].

At the time of its discovery, *H. pylori* was considered an extracellular bacterium that mainly colonized the gastric mucus layer or attached to gastric epithelial cells. However, it has since been demonstrated that *H. pylori* invades the lamina propria and gastric epithelial cells [52]. Therefore, *H. pylori* might be a target of phagocytosis by MBL activation. There have been few clinical studies regarding the role of MBL in *H. pylori* infection. Various microorganisms such as *H. pylori, Neisseria meningitidis* groups B and C, *Nocardia farcinica*, and *Legionella pneumophila* induce MBL activity in vitro [28]. Activated complements are found in the epithelium of patients with *H. pylori-*associated gastritis [29]. One pediatric study reported that *MBL2* mRNA expression in gastric biopsy specimens was higher in *H. pylori*-positive chronic gastritis than in *H. pylori*-negative chronic gastritis patients [53]. However, the study had two weaknesses in terms of its ability to reach conclusions regarding the role of *MBL2* expression in the development of *H. pylori*-infected chronic gastritis. The first weakness is the small number of biopsy specimens that were obtained, with only five *H. pylori-*positive children and four control children included. The second weakness is that they could not find any association between *MBL2* genotype and the risk of *H. pylori*-infected chronic gastritis.

The association between the *MBL2* haplotype and the risk of GC has been studied previously [30, 31]. A study conducted in Southern Italy found that the HYP + D haplotype (H/Y promoter region mutation + P untranslated region mutation + codon 52 mutation) may be a genetic marker for *H. pylori*-positive GC risk [30]. Another study performed in Warsaw, Poland found that the HY + D haplotype (H/Y promoter region mutation + codon 52 mutation) was related to an increased risk of GC compared with the HY+ A haplotype (H/Y mutation + codon 52 wild type) [31]. Therefore, the codon 52 D variant (cysteine > arginine) was specifically related to the risk of GC in two populations. In contrast to the above studies, which reported positive associations, Australian researchers evaluated healthy individuals for *H. pylori* infection, *MBL2* genotype, mannan binding level, and complement 4 level in plasma, and found that MBL deficiency, defined by either genotype or plasma activity, was not associated with higher susceptibility to *H. pylori* infection [33]. In a Japanese study, they could no significant differences were found in *MBL2* genotypes between GC patients and healthy controls [32]. Instead, the investigators found that the *MBL2* codon 54 polymorphism was weakly associated with severe AG and advanced GC [32, 54]. In the present study, we first demonstrated that the codon 54 polymorphism did not increase susceptibility to *H. pylori* infection in a Korean population. Secondly, we did not find any evidence of a role for *MBL2* in the development of gastroduodenal diseases. Thirdly, we did not find a higher risk of advanced GC or severe AG compared to early GC or mild AG, respectively, associated with *MBL2* genotype.

With regard to interracial differences, the Korean population differs from the European (Italian and Polish) and African populations reported previously. However, the results for the Korean population are very similar to those reported for the Chinese and Japanese populations [35, 38–40]. The frequencies of point mutations in European populations are in between those of the East Asian and African populations.

In the present study, serum levels of MBL, an indicator of the functional activity of MBL, differed significantly according to the genotype. However, serum MBL levels were not significantly different between the control and disease groups, because the frequency of each genotype was similar in these groups.

*H. pylori* infection stimulates *IL-8* gene expression and increases the IL-8 cytokine level in gastric epithelial cells. A significant correlation between a high level of IL-8 in the gastric mucosa and the risk of GC has been reported [13]. Our previous study found that the IL-8 level in gastric mucosal tissues was significantly higher in *H. pylori*-infected subjects compared with that in *H. pylori* non-infected subjects, irrespective of their gastroduodenal disease phenotype. After *H. pylori* eradication, the IL-8 level decreased dramatically, to the same level observed in non-infected subjects [55]. In this study, we confirmed once again that the IL-8 level in gastric mucosal tissues is mainly dependent on *H. pylori*-positive status.

It has been reported that the *IL-8* -251 T > A polymorphism is related to higher levels of IL-8 and to an increased risk of AG, gastric ulcer, and GC [13, 14]. In this study, we also demonstrated that the *IL-8 -*251 T > A polymorphism increased IL-8 production, and was significantly associated with the risk of GC and severe AG. However, many other epidemiological studies have reported negative associations between the *IL-8* -251 polymorphism and GC risk (18–23), and a meta-analysis revealed no overall association (24). In this study, we analyzed large-scale raw data from controls and GC patients from Korean, Japanese, Chinese, and Caucasian (Poland, Finland, and Portugal) populations (13–23). Korean results, including ours, were consistent with Japanese results, but not with Chinese or Caucasian results. The concordance between the Korean and Japanese results might be explained by genetic similarities. In a large study of single nucleotide polymorphism (SNP) maps covering the human genome performed in African Americans, Asians (Japanese-Chinese-Korean), and European Americans (Caucasians) [56], SNP differences in autosomes were only 5.86% between Korean and Japanese populations. Therefore, the Korean population is very similar to the Japanese population with respect to the pattern of SNPs [56].

## Conclusions

The *MBL2* codon 54 G > A polymorphism does not influence susceptibility to *H. pylori* infection and does not increase the risk of gastroduodenal diseases. We suggest that a combination of the *IL-8* -251 T > A polymorphism and increased IL-8 production in response to *H. pylori* infection may be a risk factor for severe AG and GC development in a Korean population.

## Abbreviations

AG: Atrophic gastritis
CI: Confidence intervals
DU: Duodenal ulcer
ELISA: Enzyme-linked immunosorbent assay
GC: Gastric cancer
*Helicobacter pylori*: *H. pylori*
Hp(−): *Helicobacter pylori*-negative
Hp(+): *Helicobacter pylori*-positive
IL-1β: Interleukin 1 beta
IL-8: Interleukin 8
MBL: Mannose-binding lectin
NAG: Non-atrophic gastritis
OR: Odds ratios
PCR: Polymerase chain reaction
RFLP: Restriction fragment length polymorphism
SNP: Single nucleotide polymorphism
TLR: Toll-like receptor
